# Establishment and validation of a prognostic model based on HRR-related lncRNAs in colon adenocarcinoma

**DOI:** 10.1186/s12957-022-02534-0

**Published:** 2022-03-09

**Authors:** Xingkui Tang, Yukun Lin, Jialin He, Xijun Luo, Junjie Liang, Xianjun Zhu, Tao Li

**Affiliations:** 1 Department of General Surgery, Guangzhou Panyu Central Hospital, Guangzhou, China; 2 Zhongshan School of Medicine, Sun Yat-sen University, Guangzhou, 510080 China

**Keywords:** Colon cancer, Homologous combination repair, Long non-coding RNAs, Prognostic model, HRD score

## Abstract

**Background:**

Colon cancer (CRC) is the second leading cause of cancer-related death, and its 5-year survival rate is very low. Homologous recombination repair (HRR) is deficient in most colon cancer. Some long non-coding RNAs (lncRNAs) participate in tumorigenesis of colon cancer through the HRR pathway. We aim to establish a prognostic model based on the HRR-related lncRNAs, expecting to provide a new strategy for precision treatment development in colon cancer.

**Methods:**

Pearson’s correlation was used to identify the HRR-related prognostic lncRNAs in the TCGA-COAD cohort. The TCGA-COAD cohort was randomized into the training set and the testing set. LASSO Cox regression was used to establish the model which was analyzed in the training set and validated in the testing set and the entire TCGA-COAD cohort. Finally, we explored the potential biological function of our model.

**Results:**

A prognostic model was established based on nineteen HRR-related lncRNAs in the training set. COAD patients were scored by the uniform formula and divided into high-risk and low-risk groups based on the median risk score. Patients with high-risk scores indicated poor prognosis in the training set, and the result was confirmed in the testing set and the entire TCGA-COAD cohort (all *p* < 0.01). Multivariable analysis suggested that our model was an independent factor for overall survival in COAD. The area under the curve (AUC) and C-index indicated that our model had better predictive efficiency than other indicators in the TCGA-COAD cohort. Functional enrichment analysis suggested that our model was associated with the MAPK pathway in COAD. Besides, our model was positively correlated with the HRD scores.

**Conclusion:**

A new prognostic model was established based on nineteen HRR-related lncRNAs which had excellent predictive efficiency on the prognosis of COAD. This prognostic model may provide a new strategy for prognostic prediction of COAD patients.

**Supplementary Information:**

The online version contains supplementary material available at 10.1186/s12957-022-02534-0.

## Background

Colon cancer is the most common digestive tract malignancy, and colon adenocarcinoma (COAD) is the most frequent subtype of colon cancer. As the second leading cause of cancer-related death, the occurrence of colon cancer in patients aged less than 50 years keeps increasing [1]. Despite the achievements in shifting patterns of risk factors, cancer prevention, earlier diagnosis through screening, and better treatment modalities of colon cancer [2], its morbidity is rapidly increasing and its 5-year survival rate is low [3]. Hence, it is essential to identify new predictors for prognosis in colon cancer and establish a prognostic stratification to improve adjuvant treatment selection helping patients avoid unnecessary toxicities without compromising outcomes.

The homologous recombination repair (HRR) pathway plays an important role in repairing highly cytotoxic double-stranded DNA breaks (DSB) and restarting stalled replication forks [4]. Numerous studies have investigated that harbor defects in components of the HRR pathway were an important cause of cancer incidence. However, cancer patients with DNA repair defects could benefit from treatments inducing double-stranded DNA breaks or replication fork arrest [5, 6]. Therefore, the deficiency of the HRR pathway in cancer has aroused the interest of researchers. In colon cancer, HRR is deficient in greater than 78% of tumors in an examination of a large cohort [7]. A study indicated that the mutations of HRR-related genes significantly increased the immune activities of COAD patients with microsatellite-stable (MSS), implying the feasibility of the HRR-mutation status as an immunotherapy response predictor in MSS COAD [8]. However, the function of HRR-related genes in colon cancer still needs to be further investigated.

Long non-coding RNA (lncRNA) is a module of RNA that is longer than 200nt in length and has limited or no capability of protein coding. Increasing studies have proved that lncRNAs were not artifacts of pervasive transcription of “junk DNA”; on the contrary, they played a crucial role in cell cycle controlling which was linked to cancer progression [9, 10]. Numerous lncRNAs have been reported to participate in the regulation of DSB repair by being involved in HRR [11]. Researchers have revealed that lncRNAs modulate DSB repair by five mechanisms [12]. Firstly, lncRNAs can modulate the transcription, translation, or posttranslational modifications of p53 resulting in modulating the activity of p53. Secondly, chromatin remodeling complexes can be recruited to the site of damage by lncRNAs. Thirdly, lncRNAs can act as decoys to sequester the negative regulators of DNA repair away from the damage sites. Besides, lncRNAs can interact with DNA repair proteins directly by acting as scaffolds. Finally, lncRNAs can also regulate the stability of DNA repair proteins by sponging miRNAs. Therefore, dysfunction of lncRNAs that participate in HRR would lead to pathological conditions such as cancer. In colon cancer, lncRNA HITT could attenuate ATM activation and HRR resulting in sensitized genotoxic treatment [13]. However, the function of a large number of HRR-related lncRNAs in colon cancer has not been elucidated.

Hence, in this study, we identified the prognostic HRR-related lncRNAs in COAD and established a prognostic model based on the prognostic HRR-related lncRNAs by using the least absolute shrinkage and selection operator (LASSO). Besides, we discussed the biological function of our model by using functional enrichment analysis, expecting to help the development of therapy in COAD.

## Methods

### Data collection and identification of HRR-related prognostic lncRNAs

The transcriptome profile and corresponding clinical information of 438 COAD patients were downloaded from the TCGA database (https://cancergenome.nih.gov/). The forty-one HRR-related genes were obtained from the Kyoto Encyclopedia of Genes and Genomes (KEGG) database (https://www.kegg.jp/kegg/), including SSBP1, RAD50, MRE11, NBN, ATM, BRCA1, BARD1, RBBP8, BRIP1, TOPBP1, ABRAXAS1, UIMC1, BABAM1, BABAM2, BRCC3, PALB2, BRCA2, SEM1, SYCP3, RPA1, RPA2, RPA3, RPA4, RAD51, RAD52, RAD51B, RAD51C, RAD51D, XRCC2, XRCC3, RAD54L, RAD54B, POLD1, POLD2, POLD3, POLD4, BLM, TOP3A, TOP3B, MUS81, and EME1. Pearson’s correlation analysis was used to identify the HRR-related lncRNAs that significantly related to greater than or equal to one of the 41 HRR genes (|Pearson *R*| > 0.3 and *p* < 0.01). Univariable analysis was applied to screen the prognostic HRR-related lncRNAs with *p*-value cutoff = 0.05. Finally, we identified 33 HRR-related lncRNAs that had prognostic value in the TCGA-COAD cohort.

### Establishment and validation of the prognostic model

Four hundred and thirty-eight COAD patients in the TCGA cohort were divided into two sets (training set and testing set) randomly. The training set was used to establish the prognostic model. LASSO Cox regression analysis [14, 15] was conducted to establish the prognostic model based on the prognostic HRR-related lncRNAs by using the R package “glmnet.” The following formula was used to calculate the risk score:

$$\mathrm{risk}\ \mathrm{score}={\sum}_{i=1}^n\mathrm{coef}\ \left(\mathrm{lncRNAi}\right)\times \mathrm{expr}\ \left(\mathrm{lncRNAi}\right)$$

To validate the predictive effect of the model, the testing set and the entire TCGA cohort were used as internal validation. All patients were scored using the unified formula and the median score was used to divide the patients into high-risk and low-risk groups. Kaplan–Meier survival analysis and log-rank test were used to evaluate the prognostic relevance of the prognostic model. The receiver operating characteristic (ROC) curve and the concordance index (C-index) were used to evaluate the predictive effect of risk scores on the prognostic of COAD patients in the TCGA cohort.

To test whether the prognostic value of our model can be affected by clinical features such as age, gender, tumor grade, and TNM stage, COAD patients in the TCGA cohort were stratified by age (> 60 years and ≤ 60 years), gender (male and female), tumor grade (stages I–II and stages III–IV), and TNM stage (T1–T2 and T3–T4). Kaplan–Meier survival analysis was used to draw the survival curves, and the significant difference of OS between high-risk and low-risk groups in each subset was evaluated by log-rank test.

### Functional enrichment analysis

Differentially expressed genes between the high-risk and low-risk groups in the entire TCGA cohort were identified by DESeq2 [16] with a false discovery rate (FDR) < 0.05 and |FC|>1.5 as the threshold value. KEGG pathway analysis was used to explore the potential mechanism of the model in COAD and performed by the “ClusterProfiler” R package [17].

### Homologous recombination deficiency (HRD) score analysis

HRD causes three characteristic genomic scar signatures. The loss of heterozygosity (LOH) was defined as the number of counts of chromosomal LOH regions shorter than the whole chromosome and longer than 15 Mb [18]. Large-scale state transitions (LST) were defined as chromosome breakpoint (change in copy number or allelic content) between adjacent regions each of at least 10 megabases obtained after smoothing and filtering shorter than 3Mb small-scale copy number variation [19]. Telomeric allelic imbalance (TAI) was defined as the number of regions with the allelic imbalance which extends to the sub-telomere but do not cross the centromere [20]. The HRD score is the sum of these scar signature scores [21]. Each of the genomic scar signatures was quantified by algorithms in R, and the HRD score of patients in the TCGA-COAD cohort was calculated as previously described [22]. Comparison of HRD scores between high-risk and low-risk groups in the entire TCGA-COAD cohort was performed.

### Statistical analysis

The Mann–Whitney and chi-squared *U* test was used to investigate categorical and quantitative data differences between different datasets or groups, respectively. Two-tailed *p* 0.05 was used to determine statistical significance. All the statistical analysis and visualization were performed with the R version 4.0.2 (Institute for Statistics and Mathematics, Vienna, Austria 4).

## Results

### Establishment of a prognostic model based on the HRR-related lncRNAs in COAD

**Fig. 1:**
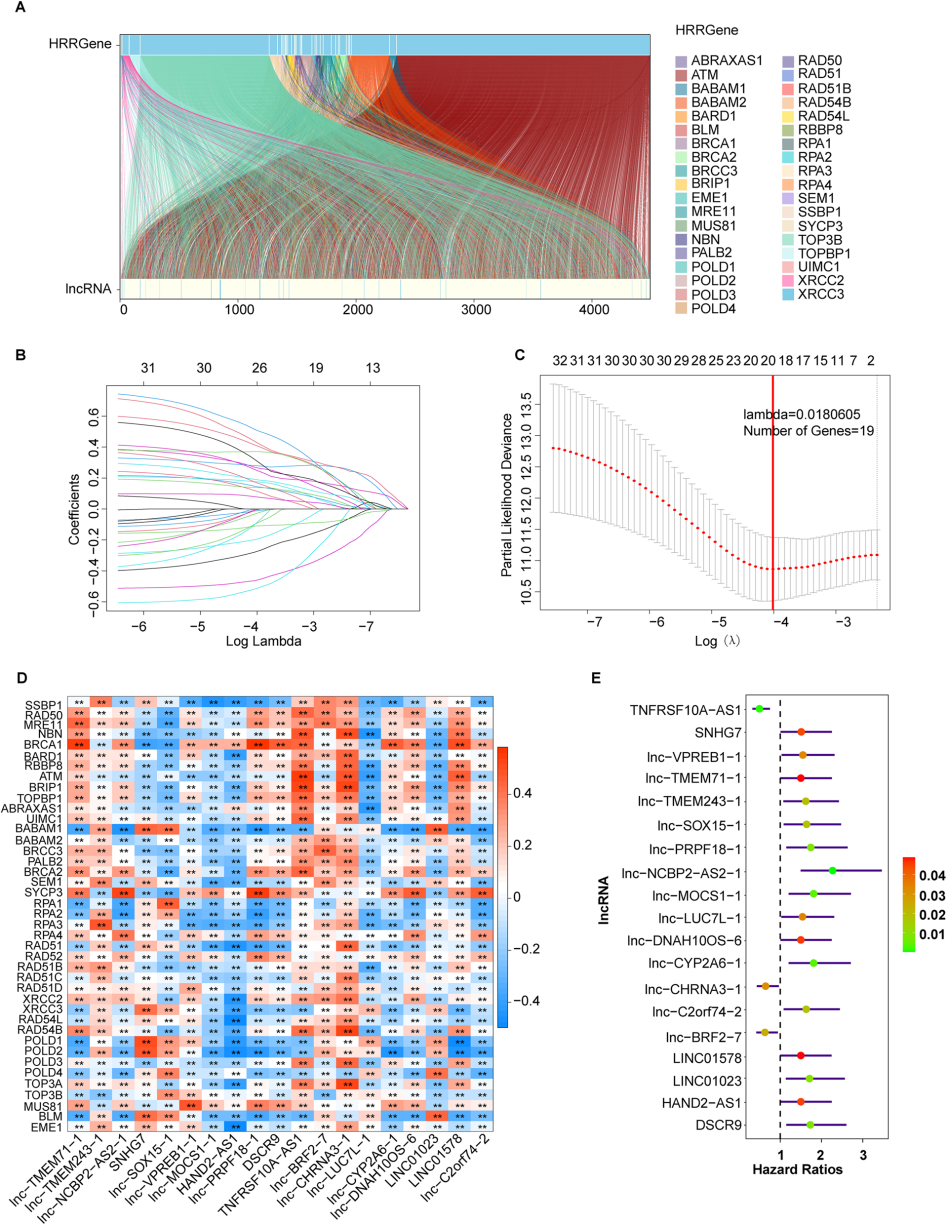
Establishment of a prognostic model based on the HRR-related lncRNAs in the TCGA training set. **A** Sankey relational diagram for 41 HRR genes and HRR-related lncRNAs. **B** The LASSO coefficient profile of 33 prognostic HRR-related lncRNAs and perpendicular imaginary lines were drawn at the value chosen by 10-fold cross-validation. **C** The tuning parameters (log l) of OS-related proteins were selected to cross-verify the error curve. According to the minimal criterion and 1-se criterion, perpendicular imaginary lines were drawn at the optimal value. **D** Heatmap for the correlations between 41 HRR genes and the 19 prognostic HRR-related lncRNAs. **E** Multivariable analysis of 19 HRR-related lncRNAs

The correlation between HRR genes and lncRNAs was displayed in the Sankey diagram (Fig. 1A, Supplementary Table 1). A total of 9216 lncRNAs were further used to select the HRR-related lncRNAs that were associated with prognosis in COAD, and 33 prognostic HRR-related lncRNAs were selected (Supplementary Table 2). The TCGA-COAD cohort was randomized into the training set and the testing set. In the training set, we established a nineteen HRR-related lncRNA prognostic model by using the LASSO Cox method, including lnc-C2orf74-2, LINC01578, LINC01023, lnc-DNAH10OS-6, lnc-CYP2A6-1, lnc-LUC7L-1, lnc-CHRNA3-1, lnc-BRF2-7, TNFRSF10A-AS1, DSCR9, lnc-PRPF18-1, HAND2-AS1, lnc-MOCS1-1, lnc-VPREB1-1, lnc-SOX15-1, SNHG7, lnc-NCBP2-AS2-1, lnc-TMEM243-1, and lnc-TMEM71-1 (Fig. 1B, C). Correlation between the nineteen lncRNAs and HRR genes showed that the selected nineteen lncRNAs were all strongly correlated with HRR genes (Fig. 1D, Supplementary Fig. 1). Besides, multivariate Cox analysis also indicated that all these nineteen HRR-related lncRNAs were independent factors on the prognosis in COAD (Fig. 1E). In addition, we also compared the expression of these nineteen HRR-related lncRNAs between the TCGA-COAD cohort and the normal samples in both the TCGA database and the Genotype-Tissue Expression (GTEx) database by using the Gene Expression Profiling Interactive Analysis (GEPIA) tool. The result showed that lnc-BRF2-7 and TNFRSF10A-AS1 were highly expressed in COAD compared to normal tissues (both *p* < 0.05), while the expression of lnc-PRPF18-1, HAND2-AS1, and lnc-MOCS1-1 was decreased in COAD compared to normal tissues (all *p* < 0.05). No significant difference was found in the expression of other HRR-related lncRNAs between COAD and normal tissues (Supplementary Fig. 2).

### Evaluation and validation of the prognostic model

**Fig. 2:**
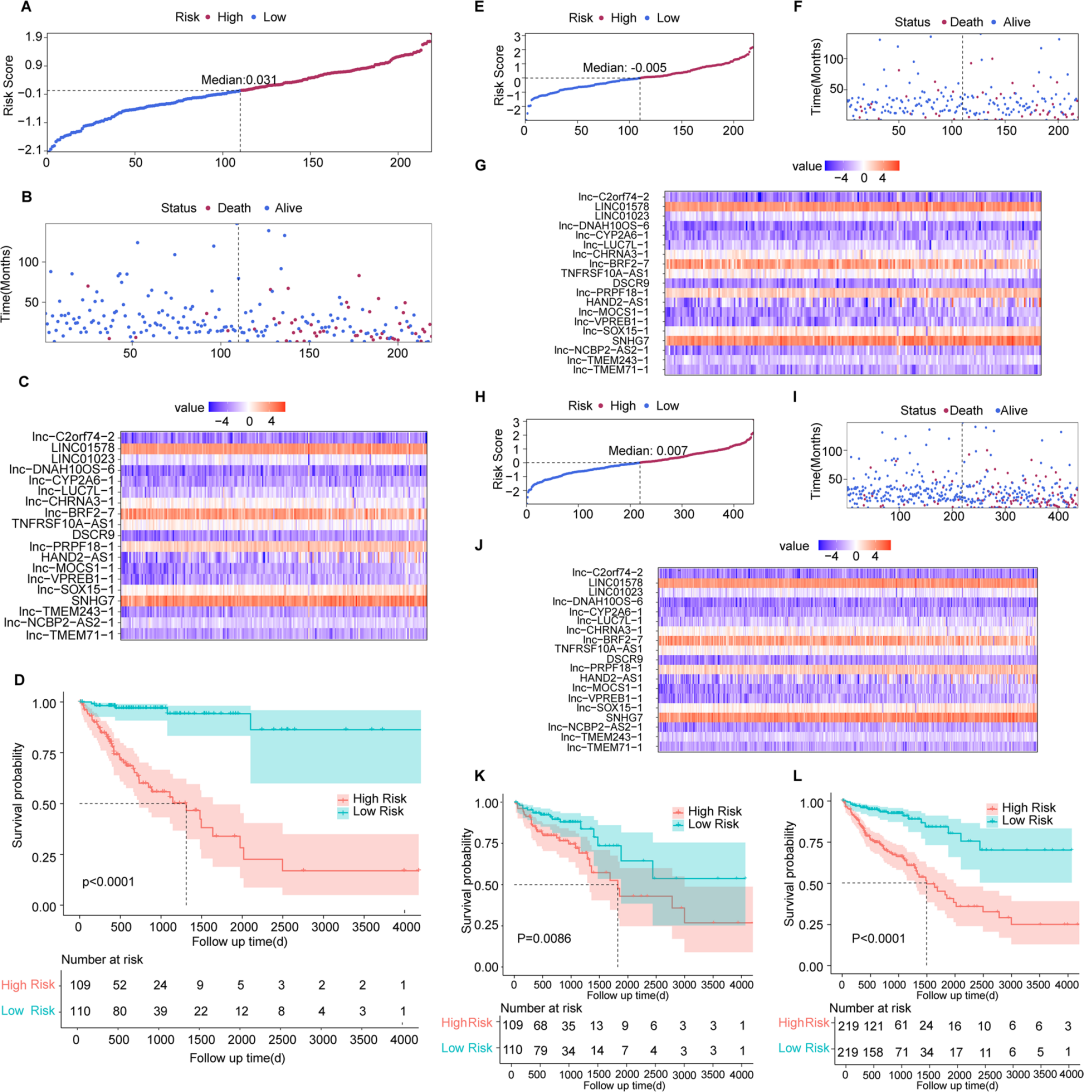
Prognostic value of the risk patterns of the 23 HRR-related lncRNAs. Distribution of HRR-related lncRNA model-based risk score in the training set (**A**), the testing set (**E**), and the entire set (**H**). Different patterns of survival status and survival time between the high- and low-risk groups in the training set (**B**), the testing set (**F**), and the entire set (**I**). Expression pattern of each patient in the training set (**C**), the testing set (**G**), and the entire set (**J**). Kaplan–Meier survival curves of the OS of patients in the high- and low-risk groups in the training set (**D**), the testing set (**K**), and the entire set (**L**)

The risk scores of COAD patients in the training set were calculated by a uniform formula, and the training set was divided into high-risk and low-risk groups according to the median risk score (Fig. 2A). The survival status of each patient is shown in Fig. 2B, which suggested that patients’ survival status deteriorated as the risk score increased. The expression standards of the 19 HRR-related lncRNAs for each patient are displayed in Fig. 2C. As shown in Fig. 2D, COAD patients with high-risk scores had significantly inferior overall survival compared with the COAD patients with low-risk scores (*p* < 0.0001). As validation, the same processes were performed in both the testing set and the entire TCGA cohort. Distribution of HRR-related lncRNA model-based risk score in the testing set and the entire TCGA cohort is depicted in Fig. 2E and H. The survival status of each patient in the testing set and the entire TCGA cohort is described in Fig. 2F and I, and the expression standards of the 19 HRR-related lncRNAs for each patient are displayed in Fig. 2G and J, respectively. Unsurprisingly, the survival status of patients in the testing set and the entire TCGA cohort was also getting worse along with the risk score increased. Similarly, the expression patterns of the 19 HRR-related lncRNAs in the testing set and the entire TCGA cohort were both consistent with that in the training set. Besides, COAD patients with high-risk scores had shorter overall survival in both the testing set (Fig. 2K, *p* = 0.0086) and the entire TCGA cohort (Fig. 2L, *p* < 0.0001), which was also consistent with the result shown in the training set.

**Fig. 3:**
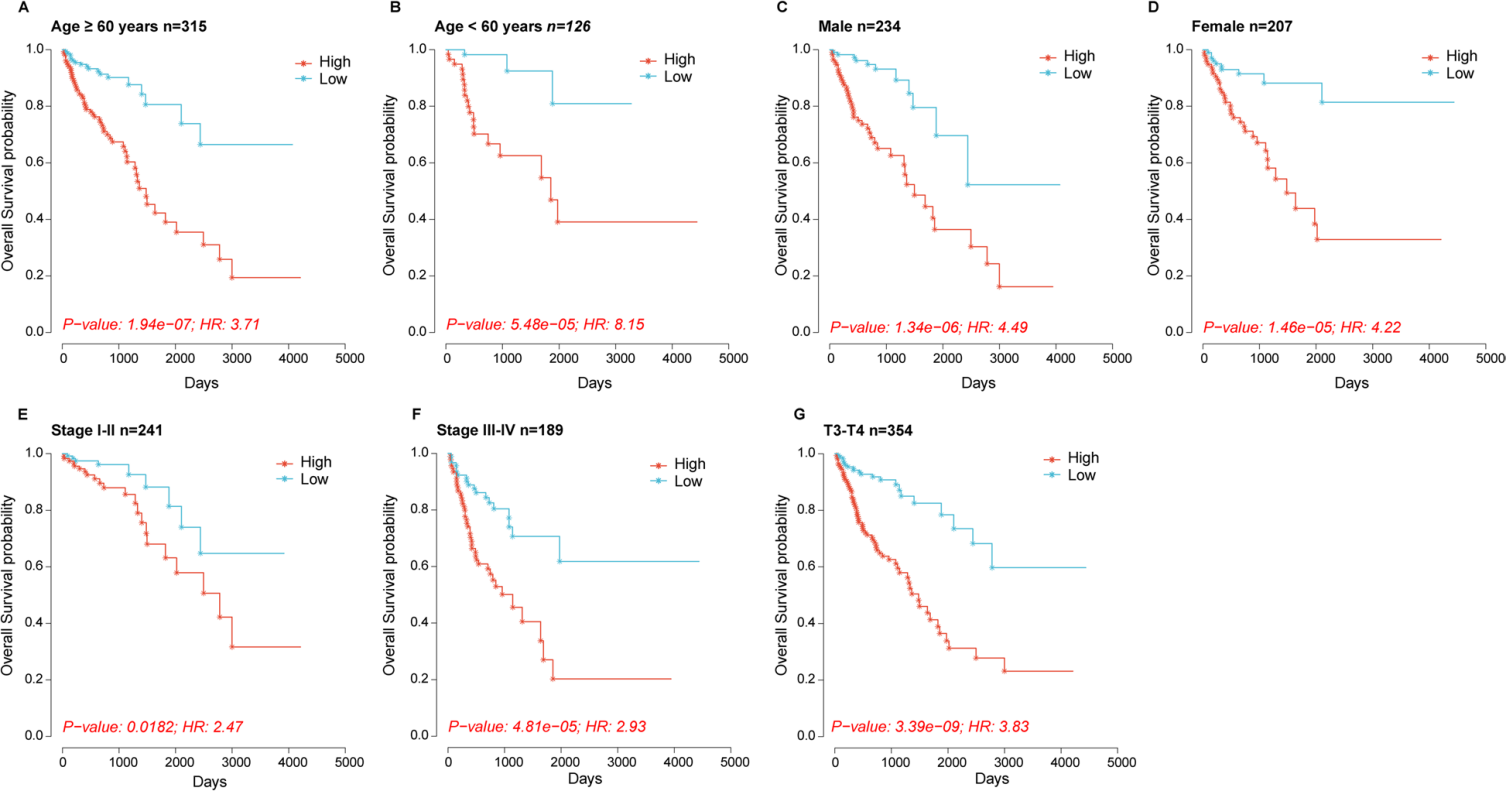
Prognostic effect of the nineteen HRR-related lncRNA prognostic model in different subgroups. **A** Kaplan–Meier survival curve of overall survival in patients older than 60 years. **B** Kaplan–Meier survival curve of overall survival in patients younger than 60 years. **C** Kaplan–Meier survival curve of overall survival in male patients. **D** Kaplan–Meier survival curve of overall survival in female patients. **E** Kaplan–Meier survival curve of overall survival in patients of stages I–II. **F** Kaplan–Meier survival curve of overall survival in patients of stages III–IV. **G** Kaplan–Meier survival curve of overall survival in patients of T3–T4 stage

And then, COAD patients in the entire TCGA cohort were stratified into different subgroups depending on the age (< 60 years and ≥ 60 years), gender (female and male), tumor stage (stages I–II and stages III–IV), and T stage (T3–T4 stage). As shown in Fig. 3, patients with lower risk scores had a better prognosis in all the subgroups (all *p* < 0.05), which indicated that the prognostic value of the nineteen HRR-related lncRNAs cannot be impacted by the different clinical features.

### Independence of the nineteen HRR-related lncRNA prognostic model

**Fig. 4:**
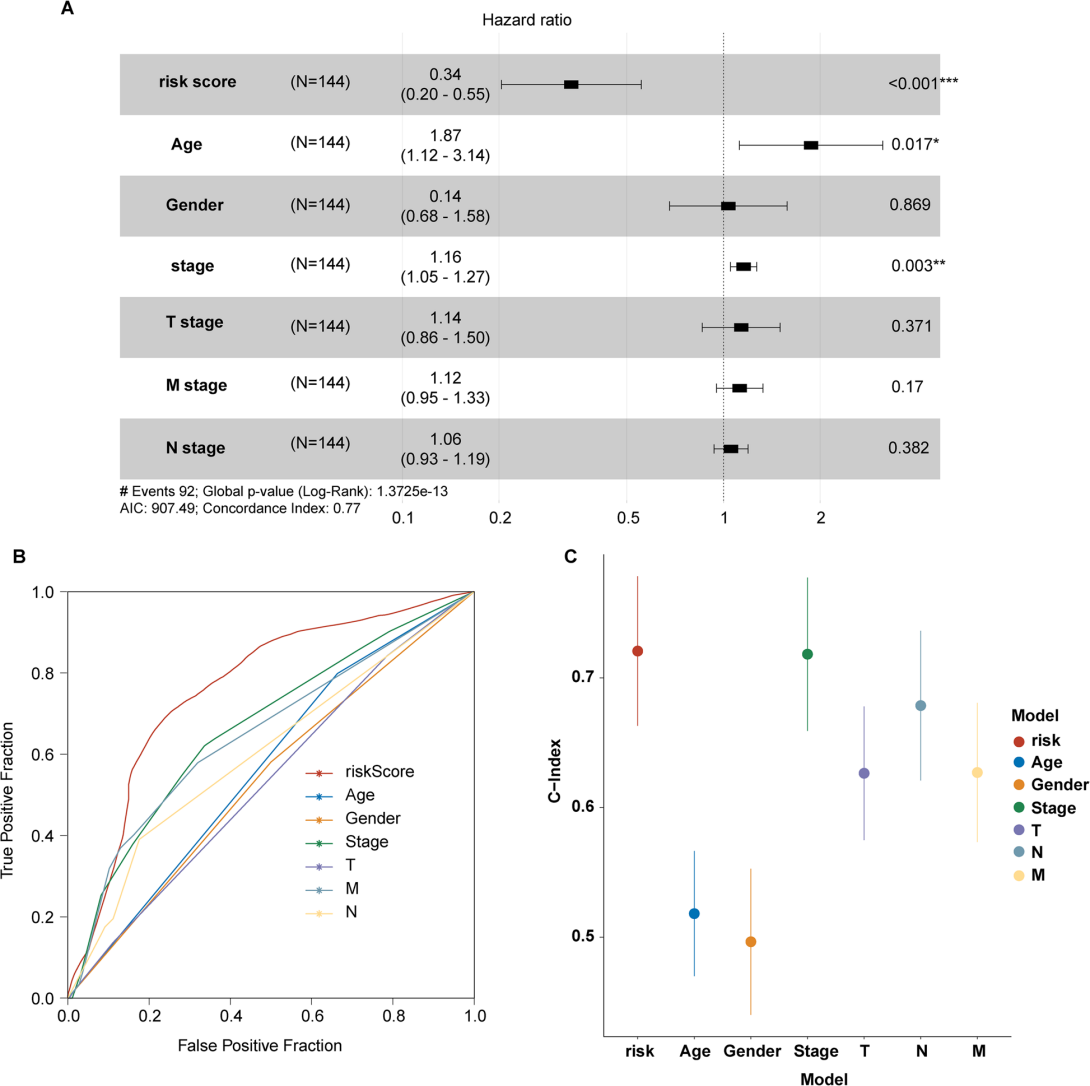
Assessment of the prognostic risk model of the HRR-related lncRNAs and clinical features in COAD. **A** Multivariate analyses of the clinical characteristics and risk score with the OS in the TCGA-COAD cohort. **B** ROC curves of the clinical characteristics and risk score on 5-year OS in the TCGA-COAD cohort. **C** Concordance indexes of the risk score and clinical characteristics in the TCGA-COAD cohort

Together with the typical features such as age, gender, tumor stage, and TMN stage, our model was an independent protective factor for OS in COAD with a hazard ratio (*HR*) = 0.34 and 95% confidence interval (95% *CI*) = 0.20–0.55 (Fig. 4A, *p* < 0.001). Besides, tumor stage was an independent prognostic factor for OS in COAD (Fig. 4A, *HR* = 1.18, 95% *CI* = 1.05–1.27, *p* = 0.003). ROC curve and C-index indicated that our model had the best predictive efficiency compared with age, gender, tumor stage, and TMN stage stratification system (Fig. 4B, C). To further evaluate the predictability of the nineteen HRR-related lncRNA prognostic model, three lncRNA-based published models were collected, including the autophagy-related lncRNA prognostic model reported by Zhou et al. [23], the immune-related lncRNA prognostic model reported by Lin et al. [24], and the genome instability-related lncRNA prognostic model reported by Yun et al. [25]. The result showed that our model had the largest AUC compared to the other three published models, which suggested that our model had better efficiency on the prediction of 2-year overall survival in COAD (Supplementary Fig. 3).

### Exploration of the biological function of the prognostic model

**Fig. 5:**
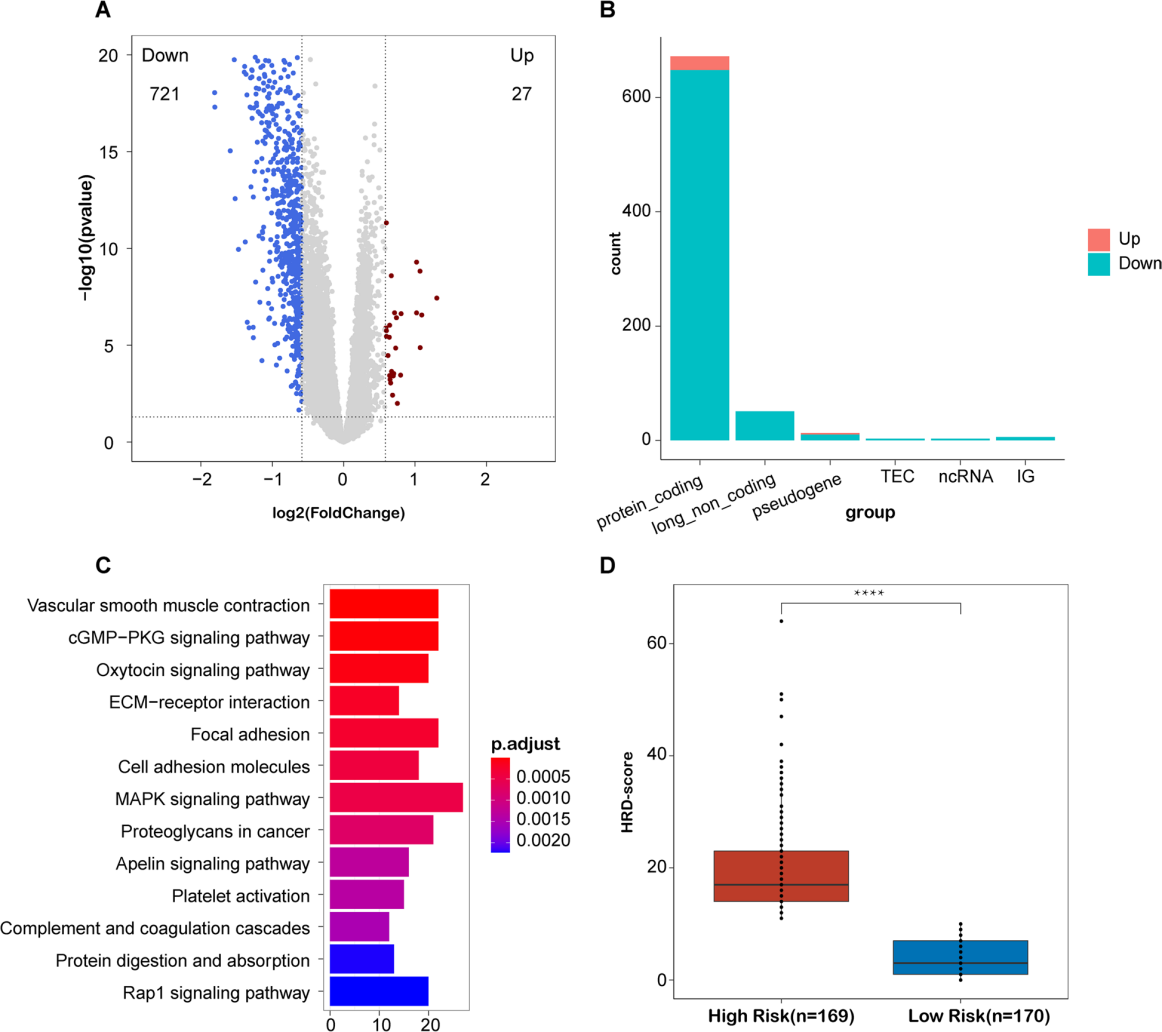
Identification of the established model-related biological processes and pathways. **A** Volcano plot of differentially expressed genes, red dots represent upregulated genes and blue dots represent downregulated genes. **B** Bar diagram of differential expressed genes, the *X*-axis is gene ontology and the *Y*-axis is the number of genes; red represents upregulated genes, and blue represents downregulated genes. **C** Functional enrichment analysis. **D** Comparison of HRD score between high and low groups in the TCGA-COAD cohort

Differentially expressed gene analysis was performed between the high-risk and low-risk groups in the TCGA-COAD cohort. As displayed by the volcano plot, 27 upregulated genes and 721 downregulated genes were identified as the differentially expressed genes (DEGs) (Fig. 5A), and the composition of these DEGs is displayed as the bar diagram in Fig. 5B. KEGG pathway analysis was performed on the DEGs and the result showed that the DEGs were majorly enriched in MAPK signaling pathway, focal adhesion, ECM-receptor interaction, and proteoglycans in cancer and platelet activation (Fig. 5C). Besides, we predicted the target genes of the HRR-related lncRNAs using the starbase database [26] and constructed the HRR-related lncRNA interaction network by using the STRING database [27]. Pathway enrichment analysis on the metascape website revealed that the HRR-related lncRNA interaction network was related to the nuclear chromosome segregation, DNA damage response, and CDC42 pathway (Supplementary Fig. 4). Additionally, we compared the HRD score between high-risk and low-risk groups in the TCGA-COAD cohort and the result showed that patients in the high-risk group had significantly higher HRD score than those in the low-risk group (Fig. 5D, *p*<0.0001), which indicated that the nineteen HRR-related lncRNA model could be the potential predictor for the response of PARP inhibitors.

## Discussion

lncRNAs are a kind of non-coding RNAs longer than 120nt in length, which have been illuminated as novel biomarkers and targets for the diagnosis and therapy in tumors [28–30]. In recent years, multiple lncRNAs have been reported to be regulators in the development of CRC and be associated with prognosis in CRC. For example, lncRNA LINC01224 regulated the proliferation and invasion of CRC through modulating the miR-485-5p/MYO6 axis [31]. Therefore, many lncRNA-based prognostic models have been developed, such as a six lncRNA risk prognosis model reported by Gao et al. [32]. However, the role of a large number of lncRNAs in CRC remains to be illuminated. In this study, we selected nineteen lncRNAs that were correlated with HRR genes and associated with prognosis in COAD to establish a prognostic model. The model had better predictive performance than other traditional indicators and recently published models in COAD.

Among these nineteen HRR-related lncRNAs, six lncRNAs have been reported including LINC01578, LINC01023 [33], TNFRSF10A-AS1, DSCR9 [34, 35], HAND2-AS1, and SNHG7. LINC01578 has been reported to be the driver in colon cancer metastasis [36]. TNFRSF10A-AS1 was related to autophagy in colorectal cancer and contributed to poor prognosis in colorectal cancer [37]. In addition, HAND2-AS1 is an important lncRNA that is significantly related to the regulation of cell proliferation, apoptosis, invasion, metastasis, and energy metabolism as well in numerous human tumors [38]. Besides, lncRNA SNHG7 is an important tumorigenesis promoter as well [39]. Additionally, other lncRNAs were unveiled for the first time. To evaluate the clinical significance of our model in COAD patients, we separated the COAD patients into high-risk and low-risk groups based on the median value of the risk score and compared the overall survival between the two groups. Unsurprisingly, patients in the high-risk group had worse prognosis than those in the low-risk group, suggesting that our model can be used to predict the prognosis of COAD patients. Moreover, to indicate whether the prognostic value of our model can be impacted by some clinical characteristics, COAD patients in the TCGA cohort were grouped by age, gender, tumor stage, and TMN stage. We found that our model had excellent prognostic effect in every subgroup. Besides, multivariate analysis also demonstrated that our model was an independent factor for prognosis in COAD. Furthermore, ROC curve and C-index indicated that our model had the best performance on the 5-year OS prediction in COAD which suggested great dependability of our model in COAD. Additionally, we also compared the predictability of our model and three recently published models and the result also suggested that our model had better predictive efficiency than other published molecular models. Together, our findings illuminated that the nineteen HRR-related lncRNA prognostic model was a reliable predictor of prognosis in COAD.

To further unveil the potential mechanism of this model in COAD, we identified the DEGs between high-risk and low-risk groups in the TCGA-COAD cohort, and the functional enrichment analysis was conducted to figure out the biological pathway of these DEGs. We indicated that our model was associated with the MAPK signaling pathway which was a famous signaling pathway that related to tumorigenesis and tumor metastasis [40]. In colon cancer, ERK/MAPK signaling pathway was involved in the effects of hepatocyte growth factor (HGF) of promoting proliferation and regulating cell cycle and apoptosis of cancer cells [41]. p38α MAPK pathway also played a predominant role in colon cancer development and chemoresistance [42]. In recent years, FDA-approved inhibitors targeting the MAPK pathway have shown promising clinical responses in patients with colorectal cancer [43]. Our findings suggested that the nineteen HRR-related lncRNA model might be the indicator for MAPK inhibitor response and provided new thought for novel biomarkers in COAD.

Numerous studies have illuminated that HRD existed in many types of cancer including ovarian cancer [44], breast cancer [45], lung cancer [46], and colon cancer [7]. HRD score is an indicator to quantify the degree of HRD in tumors, and it has been employed on prediction of treatment response in triple-negative breast cancer [47, 48]. The presence of HRD results in irreparable DNA damage from platinum-containing drugs, which leads to cell death; therefore, tumors with HRD were more sensitive to PARP inhibitors such as Olaparib [49]. Therefore, we compared the HRD scores between high-risk and low-risk groups in the TCGA-COAD cohort. We suggested that our model was positively correlated with the HRD score in the TCGA-COAD cohort. Our findings indicated that our model had the potential to be the predictor for PARP inhibitor response and provided new ideas for precision treatment in COAD.

In conclusion, we established a prognostic model containing nineteen HRR-related lncRNAs in COAD and its predictive efficiency of OS was excellent. Besides, our model also had a wonderful performance in the prediction of 5-year OS compared with other predictive factors in the TCGA-COAD cohort. Additionally, we considered that our model could be the potential predictor and therapeutic target in the precision treatment of COAD.

However, the current study also has some limitations. We established and evaluated the model based on the published datasets and our study was more suitable for retrospective analysis. We preliminarily unveiled the mechanism of our model in COAD and further validation needs to be investigated. In the future, these shortcomings may be overcome through prospective experiments with a large number of patients.

## Conclusion

We established a new prognostic model based on the HRR-related lncRNAs in COAD, and the model had excellent predictive efficiency. Besides, the model had better performance than other traditional indicators and recently published models. Besides, we preliminary discussed the potential function of the model in COAD and hope to provide new ideas for the clinical treatment of COAD.

## Supplementary Information

**Additional file 1: Supplementary Figure 1.** Coexpression network between the nineteen HRR-related lncRNAs and HRR genes.

**Additional file 2: Supplementary Figure 2.** Comparison of the expression of the nineteen HRR-related lncRNAs between the COAD and normal tissues.

**Additional file 3: Supplementary Figure 3.** Comparison of ROC curve between the nineteen HRR-related lncRNAs prognostic model and other three published models in the TCGA-COAD cohort.

**Additional file 4: Supplementary Figure 4.** HRR-related lncRNAs interaction network construction and pathway enrichment analysis. (A) Interaction network of HRR-related lncRNAs and its target genes. (B) Pathway enrichment analysis.

**Additional file 5: **Supplementary tables.

## Data Availability

The datasets generated and/or analyzed during the current study are available in the TCGA repository, https://cancergenome.nih.gov/.

## Abbreviations

CRC: Colon cancer
COAD: Colon adenocarcinoma
HRR: Homologous recombination repair
lncRNAs: Long non-coding RNAs
TCGA: The Cancer Genome Atlas
LASSO: Least absolute shrinkage and selection operator
ROC: Receiver operating characteristic curve
KEGG: Kyoto Encyclopedia of Genes and Genomes
C-index: Concordance index
HRD: Homologous recombination deficiency
LOH: Loss of heterozygosity
LST: Large-scale state transitions
TAI: Telomeric allelic imbalance
