# Supplementary figures and images for: Establishment and validation of a prognostic model based on HRR-related lncRNAs in colon adenocarcinoma
Source: World J Surg Oncol. 2022 Mar 9;20:74. doi: 10.1186/s12957-022-02534-0 (PMC8905762; doi:10.1186/s12957-022-02534-0)

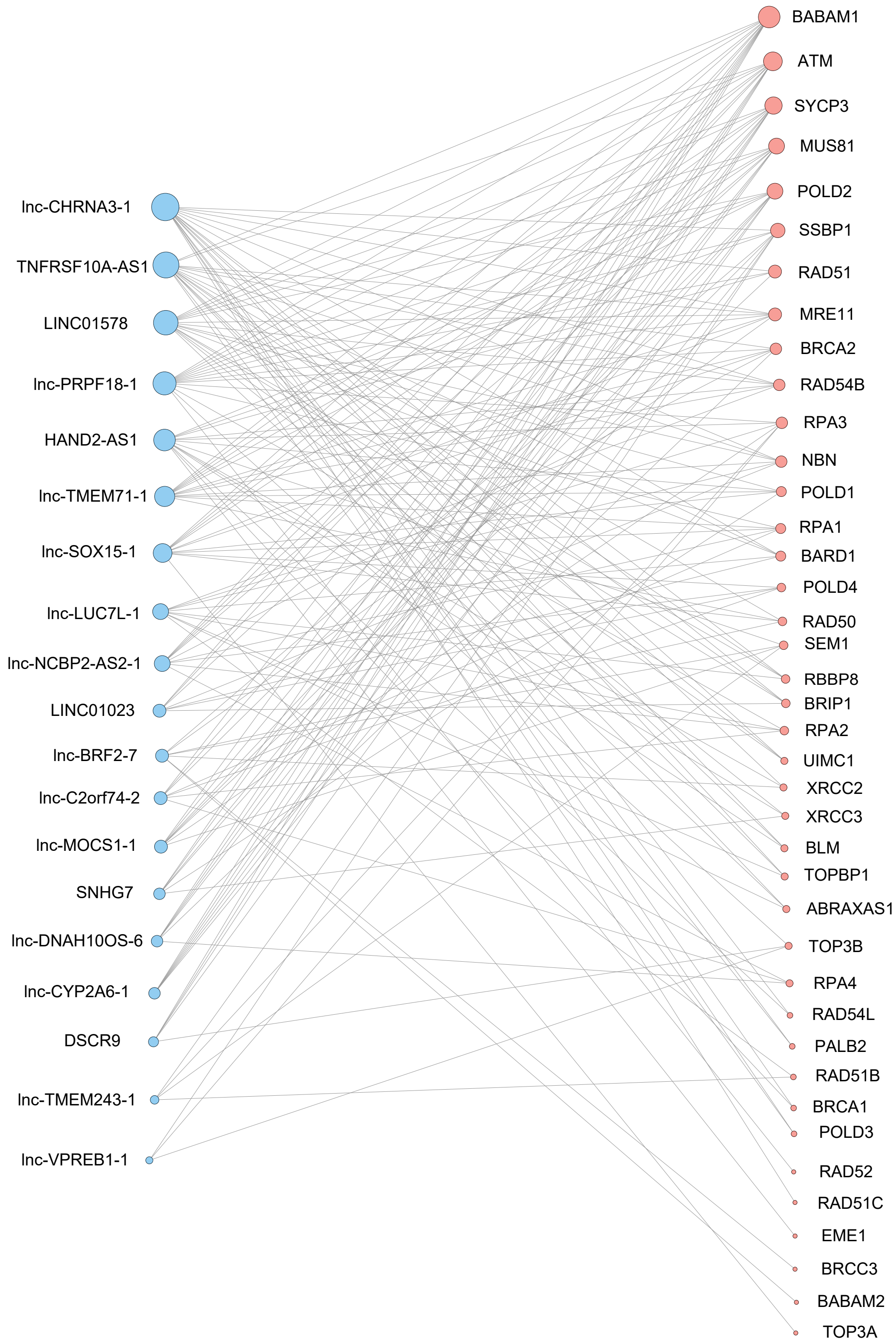

Supplement: Supplementary file 1 — Additional file 1: Supplementary Figure 1. Coexpression network between the nineteen HRR-related lncRNAs and HRR genes. [file 12957_2022_2534_MOESM1_ESM.pdf]

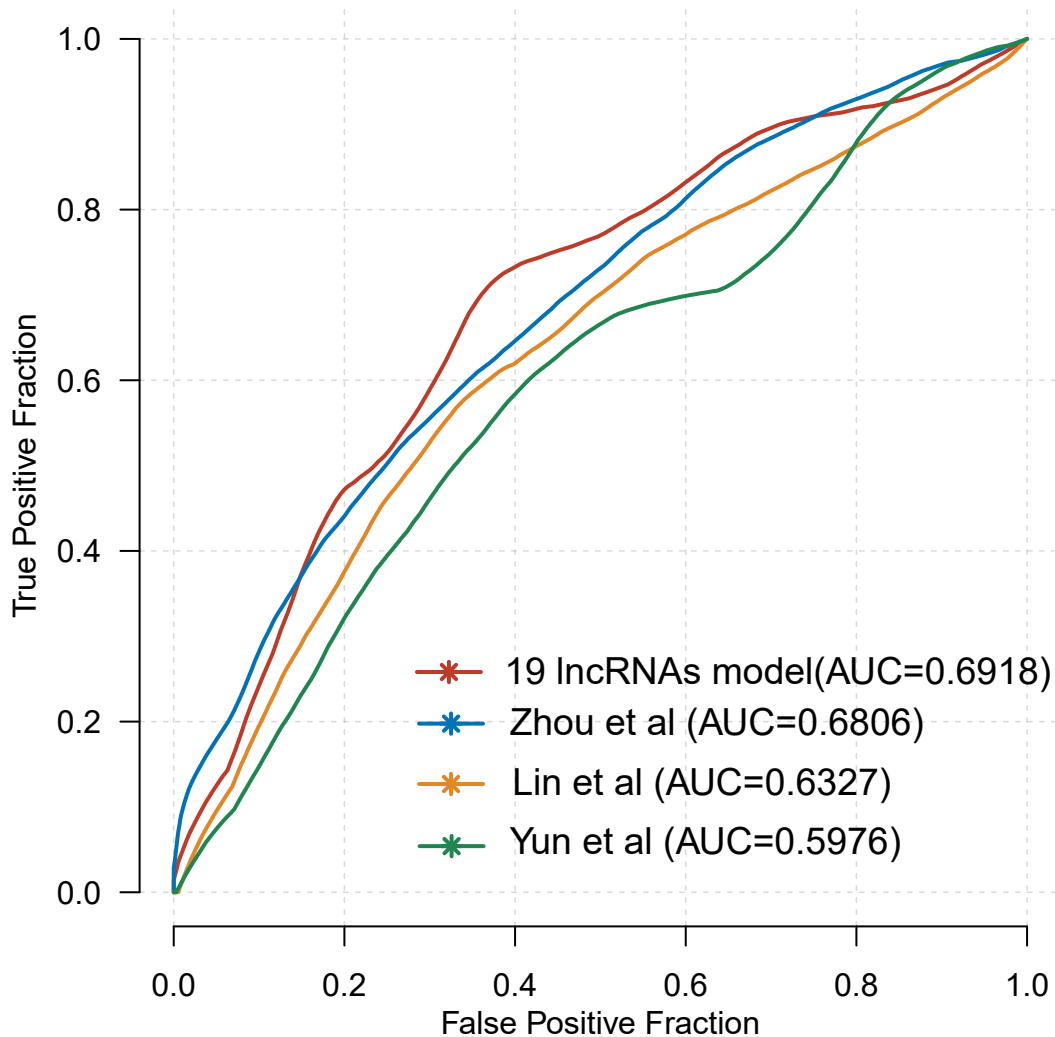

Supplement: Supplementary file 3 — Additional file 3: Supplementary Figure 3. Comparison of ROC curve between the nineteen HRR-related lncRNAs prognostic model and other three published models in the TCGA-COAD cohort. [file 12957_2022_2534_MOESM3_ESM.pdf]

A

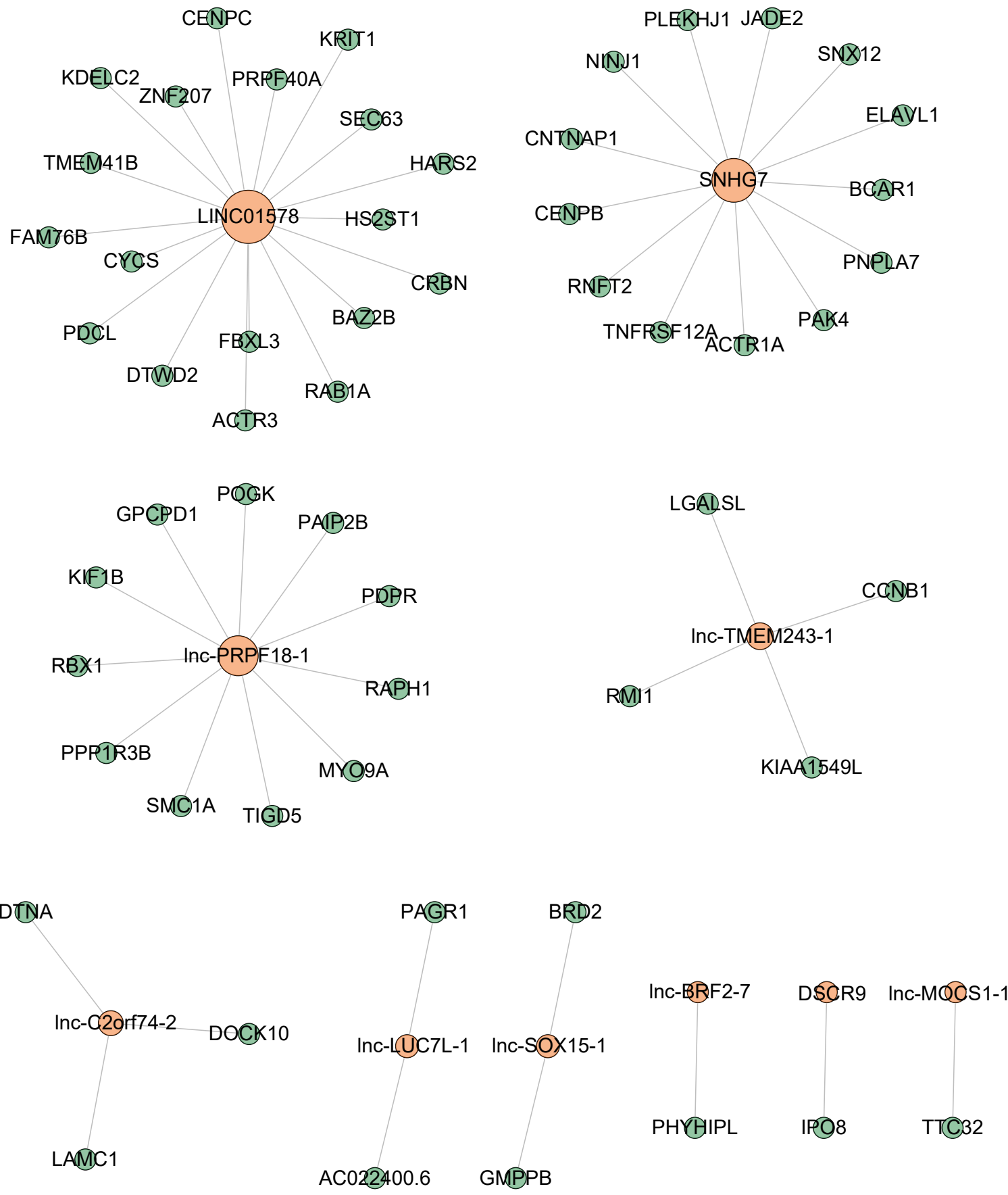

B

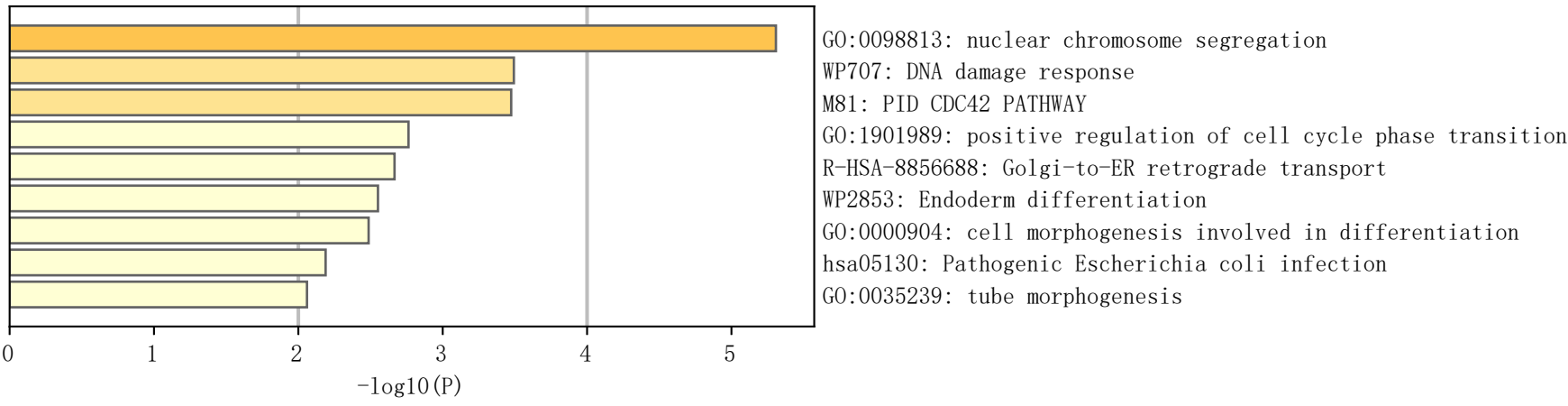

Supplement: Supplementary file 4 — Additional file 4: Supplementary Figure 4. HRR-related lncRNAs interaction network construction and pathway enrichment analysis. (A) Interaction network of HRR-related lncRNAs and its target genes. (B) Pathway enrichment analysis. [file 12957_2022_2534_MOESM4_ESM.pdf]
