# Supplementary material for: Establishment and validation of a prognostic model based on HRR-related lncRNAs in colon adenocarcinoma
Source: World J Surg Oncol. 2022 Mar 9;20:74. doi: 10.1186/s12957-022-02534-0 (PMC8905762; doi:10.1186/s12957-022-02534-0)
Supplement: Supplementary file 2 — Additional file 2: Supplementary Figure 2. Comparison of the expression of the nineteen HRR-related lncRNAs between the COAD and normal tissues. [file 12957_2022_2534_MOESM2_ESM.pdf]

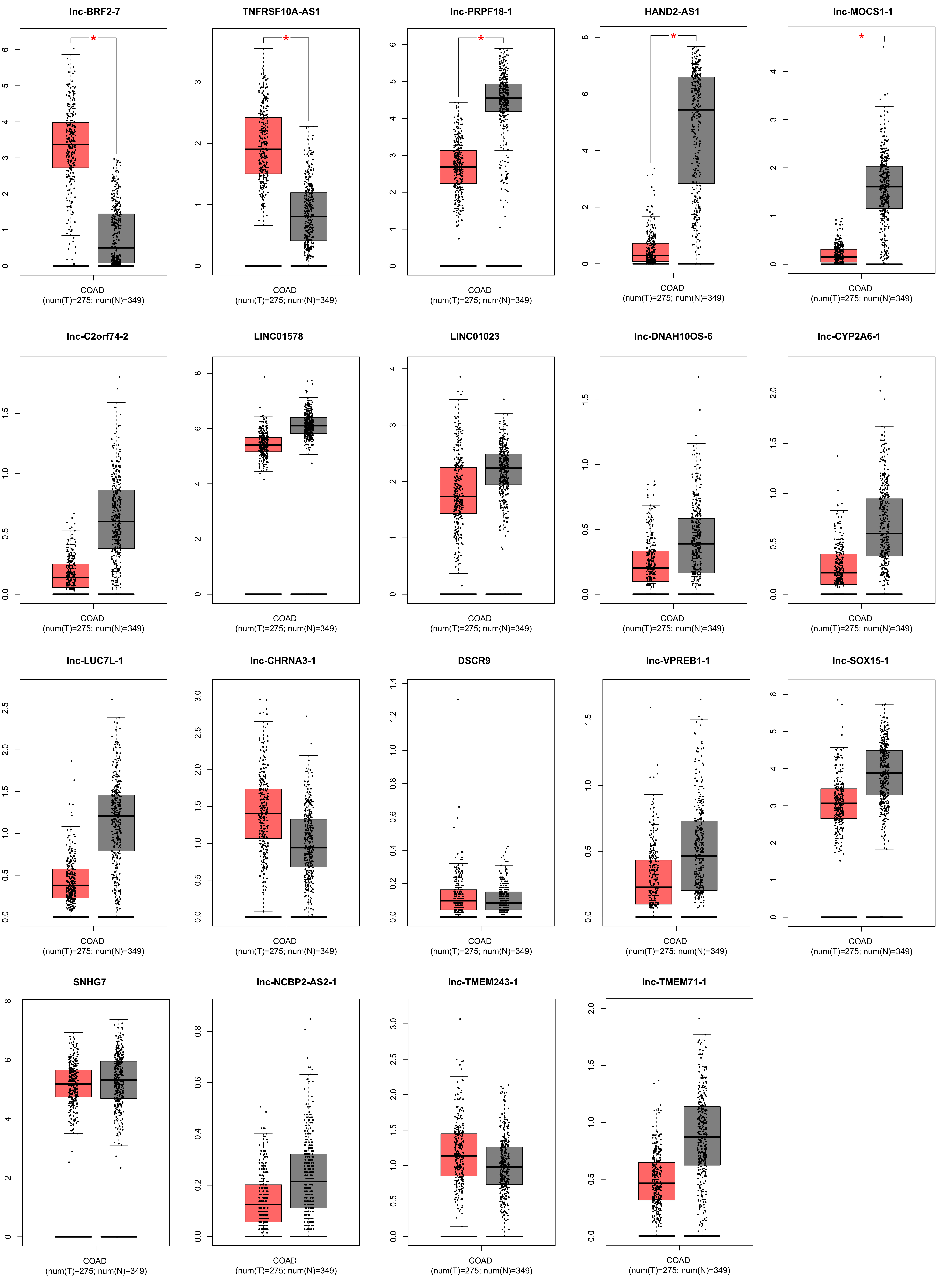
lnc-BRF2-7
TNFRSF10A-AS1
lnc-PRPF18-1
HAND2-AS1
lnc-MOCS1-1
lnc-C2orf74-2
LINC01578
LINC01023
lnc-DNAH10OS-6
lnc-CYP2A6-1
lnc-LUC7L-1
lnc-CHRNA3-1
DSCR9
lnc-VPREB1-1
lnc-SOX15-1
SNHG7
lnc-NCBP2-AS2-1
lnc-TMEM243-1
lnc-TMEM71-1
COAD
(num(T)=275; num(N)=349)
